# Altered B Cell Homeostasis and Toll-Like Receptor 9-Driven Response in Type 1 Diabetes Carriers of the C1858T *PTPN22* Allelic Variant: Implications in the Disease Pathogenesis

**DOI:** 10.1371/journal.pone.0110755

**Published:** 2014-10-21

**Authors:** Elena Gianchecchi, Antonino Crinò, Ezio Giorda, Rosa Luciano, Valentina Perri, Anna Lo Russo, Marco Cappa, M. Manuela Rosado, Alessandra Fierabracci

**Affiliations:** 1 Autoimmunity Laboratory, Immunology and Pharmacotherapy Area, Bambino Gesù Children’s Hospital, IRCCS, Rome, Italy; 2 Division of Endocrinology, Bambino Gesù Children’s Hospital, IRCCS, Rome, Italy; 3 B cell Development Laboratory, Immunology and Pharmacotherapy Area, Bambino Gesù Children’s Hospital, IRCCS, Rome, Italy; 4 Research Laboratories, Bambino Gesù Children’s Hospital, IRCCS, Rome, Italy; University of Michigan Medical School, United States of America

## Abstract

Type 1 diabetes is an autoimmune disease caused by the destruction of pancreatic beta cells by autoreactive T cells. Among the genetic variants associated with type 1 diabetes, the C1858T (Lyp) polymorphism of the protein tyrosine phosphatase non-receptor type 22 (*PTPN22*) gene alters the function of T cells but also of B cells in innate and adaptive immunity. The Lyp variant was shown to diminish interferon production and responses upon Toll-like receptor stimulation in macrophages and dendritic cells, possibly leading to uncontrolled infections as triggers of the diabetogenic process. The aim of this study was to unravel the yet uncharacterized effects that the variant could exert on the immune and autoimmune responses, particularly regarding the B cell phenotype, in the peripheral blood lymphocytes of diabetic patients and healthy controls in basal conditions and after unmethylated bacterial DNA CpG stimulation. The presence of the Lyp variant resulted in a significant increase in the percentage of transitional B cells in C/T carriers patients and controls compared to C/C patients and controls, in C/T carrier patients compared to C/C controls and in C/T carrier patients compared to C/C patients. A significant reduction in the memory B cells was also observed in the presence of the risk variant. After four days of CpG stimulation, there was a significant increase in the abundance of IgM^+^ memory B cells in C/T carrier diabetics than in C/C subjects and in the groups of C/T carrier individuals than in C/C individuals. IgM^−^ memory B cells tended to differentiate more precociously into plasma cells than IgM^+^ memory B cells in heterozygous C/T subjects compared to the C/C subjects. The increased Toll-like receptor response that led to expanded T cell-independent IgM^+^ memory B cells should be further investigated to determine the putative contribution of innate immune responses in the disease pathogenesis.

## Introduction

Protein tyrosine phosphatase non-receptor type 22 (PTPN22), also termed lymphoid tyrosine phosphatase (Lyp) and encoded by *PTPN22*, and its murine ortholog proline enriched phosphatase (PEP) are preferentially expressed in hematopoietic and immune cells [1], the T and B cell compartments, natural killer cells (NK) and dendritic cells (DCs), monocytes and macrophages [2].

Intensive investigation was propelled in recent years by the discovery that a variant form of Lyp produced by a single nucleotide polymorphism (SNP) at the position 1858 (C1858T) that causes the substitution of the amino acid residue 620 from arginine (R) to tryptophan (W) in exon 14 of the Lyp protein (Lyp R620W) [2]–[6] is strongly associated with several autoimmune conditions [7]–[14]. In particular the variant allele C1858T represents a genetic risk factor for insulin-dependent diabetes (type 1 diabetes, T1D) [3], [8].

A preponderance of experimental evidence suggests that the C1858T polymorphism is a gain-of-function mutation [3], [4], [7] and that it induces a higher negative regulation of the T cell receptor (TCR) signaling [7], [15], [16] by encoding a more active phosphatase [3], [7]. The Lyp variant could contribute to subtle TCR signaling defects by acting on the zeta-chain associated protein 70 kDa (ZAP-70) and on CD3 zeta chains. This may have important implications in thymocyte tolerization and the escape of autoreactive T lymphocytes through the positive selection of otherwise negatively selected autoimmune T cells [17] and by decreasing differentiation into regulatory rather than effector T cells [18].

More recently, the effect of the Lyp variant was shown to extend beyond T cells because individuals heterozygous for the C1858T variant of the *PTPN22* gene were found to exhibit an altered B cell compartment. Arechiga [19] and, in particular Rieck [15], demonstrated a reduction in memory (CD19^+^CD27^+^) B cells in subjects harboring this variant. Furthermore, memory B cells, when appropriately challenged, showed a diminished B cell receptor (BCR) signaling response and resistance to apoptosis. Increased survival was also observed in both transitional and naïve B cells. The effect on the generation of this memory B cell population appeared not to be related to the altered T cell function and was instead due to the direct influence of the *PTPN22* genetic variant on B cell activation. Habib [20] further observed that alterations in the transitional, mature and memory B cell subsets were associated with the variant in healthy subjects and resulted in increased transitional and anergic autoreactive B cells (CD19^+^CD27^−^IgD^+^IgM^−^). These alterations in the composition of the B cell pool were also characteristic of nearly all T1D subjects, irrespective of the *PTPN22* genotype. Functionally impaired proximal BCR signaling was observed in both naïve and memory B cells from T1D subjects, as were parallel homeostatic alterations in the periphery (i.e., increased transitional and naïve autoreactive B cells and decreased memory B cells).

Menard and colleagues [21] confirmed the presence of a higher number of polyreactive new emigrant/transitional [CD19^+^CD21^low^CD10^+^IgM^high(hi)^CD27^−^] and mature/naïve B cells (CD19^+^CD21^+^CD10^−^IgM^+^CD27^−^) in healthy controls carrying the polymorphism than in non-carrier controls, confirming the role of the *PTPN22* variant in the induction of altered central and peripheral B cell tolerance mechanisms. In addition, T1D patients with the variant were characterized by higher frequencies of autoreactive clones in the new emigrant/transitional and mature naïve B cell compartments, similar to those observed in C1858T healthy donors. Moreover, the presence of the SNP induced an up-regulation of several genes belonging to the BCR, CD40 and Toll-like receptor (TLR) signaling pathways that converge on nuclear factor-kB (NF-kB), indicating B cell activation [21].

The impaired central and peripheral B cell tolerance mechanisms observed in T1D were similar to those reported in Rheumatoid Arthritis (RA) and Systemic Lupus Erythematosus (SLE) patients [21], [22] carrying the C1858T polymorphism. Similar defects were observed in healthy subjects carrying the variant before the clinical onset of autoimmunity.

The effects of the Lyp variant were shown on both innate and adaptive immune responses, and it was recently discovered that Lyp positively regulates type 1 IFN production in myeloid cells upon TLR engagement [23]. *Ptpn22* promotes type 1 IFN-dependent processes *in vivo*, including innate and adaptive cellular responses to viral infection, and suppresses inflammation in colitis and arthritis. Wang and colleagues [23] further demonstrated that the human Lyp variant diminished IFN production and IFN responses upon TLR stimulus in macrophages and dendritic cells. As confirmed by Ivashkiv [24], in disorders such as T1D, diminished IFN production can compromise host defenses and allow for the development of uncontrolled infections that ultimately result in autoimmunity triggers.

The relative contribution of the Lyp variant to the development of immune responses and the pathogenesis of T1D remains to be fully elucidated. There is compelling evidence that adaptive responses and abnormalities in antigen presentation are the primary drivers of pathogenesis in T1D [25]. The pathophysiology is most likely due to the presentation of beta cell antigens to T lymphocytes within the lymph nodes. Autoreactive T cells then migrate to the pancreas where beta cell destruction occurs. Autoantibodies are predictors of the disease onset in the preclinical period, making the role of B cells enigmatic [25]–[28]. B cells may play a crucial role as antigen presenting cells. Recent rituximab trials have showed that the treatment is able to delay the progression of disease [29] by attenuating beta cell loss without affecting the proliferative response to beta cell antigens [30]. In non-obese diabetic (NOD) mice, recent evidence suggest that in addition to the contribution of TLRs in eliminating pathogens, altered innate immune mechanisms may elicit aggressive autoimmune responses in the ongoing insulitis [31]. Furthermore, TLR9-deficient NOD mice are protected from disease, and a putative role for antibodies of the IgM isotype spontaneously secreted by B1a B cells with a low threshold for innate immune activation has been demonstrated [31]–[34].

In this study, we investigated the effects of the Lyp variant on the immune and autoimmune responses, particularly regarding the peripheral blood B cell function, in T1D patients and healthy controls following CpG-induced stimulation. CpG mimics a TLR9 stimulus to most immature B cell types, i.e. transitional B cells, that consequently acquire the phenotype of IgM^+^ memory B cells before terminally differentiating into plasma cells. These cells generate the repertoire of antibacterial natural antibodies for protection at birth [35]. In the analysis of B cell subsets transitional B cells are identified as CD24^hi^CD38^hi^CD27^−^, mature/naïve B cells as CD24^hi^CD38^−^CD27^−^ [36]. In the differentiation process, since the CD24 is lost, switched memory cells are identified as CD19^+^CD27^+^IgM^−^, IgM^+^ memory B cells as CD19^+^CD27^+^IgM^+^ and plasma cells as CD19^+^CD27^hi^IgM^+^ and CD19^+^CD27^hi^IgM^−^ [35].

## Materials and Methods

### Subjects

The patient group consisted of 26 T1D patients who were referred from the Department of Endocrinology at Bambino Gesù Children’s Hospital (OPBG) during the past 2 years.

The patients’ sera were tested for autoantibodies (Abs) to glutamic acid decarboxylase (isoform 65) (GADA), protein tyrosine phosphatase insulinoma associated antigen 2 (IA2) and insulin (IAA) by radioimmunoassay (RIA), to thyroglobulin (Tg), thyroperoxidase (TPO) and transglutaminase (tTGA) by chemiluminescence (ADVIA Centaur analyzer, Siemens Healthcare, Germany) and to parietal cells (PCA), the adrenal cortex (ACA) and islet cells by indirect immunofluorescence (IFL). The control group included 45 healthy donors recruited from the Blood Transfusion Centre at OPBG; they had no history of autoimmunity, and no autoantibodies were detected in their sera.

All control subjects were matched for sex, age, ethnic origin and geographical area. All enrolled patients and controls were unrelated. All subjects were enrolled in the investigation after obtaining written informed consent. The study was approved by the local Institutional Review Board (IRB) of Bambino Gesù Children’s Hospital, regulating the use of human samples for experimental studies. The informed consent for children was obtained from the next of kin. Consent on behalf of the children was written. Participant consent was recorded using a paper-based inventory system. The IRB approved the consent procedure.

### Cell preparation

Peripheral blood mononuclear cells (PBMC) were separated by Ficoll-Hypaque (Histopaque, Sigma-Aldrich Chemical C, St Louis, MO, USA) from 5–10 mL of sodium heparinized venous blood samples, washed twice in phosphate buffered saline (PBS) (Lonza, Verviers, Belgium), and frozen in liquid nitrogen.

### Detection of the C1858T variant in the *PTPN22* gene

Molecular analysis of the C1858T (R620W) polymorphism of the autoimmunity predisposing gene *PTPN22* [37] was evaluated in the DNA of patients and controls using a XcmI restriction fragment length polymorphism-PCR (polymerase chain reaction) method.

### Stimulation of PBMC with CpG and the proliferation assay

Before stimulation, PBMC were labeled with 5-chloromethylfluorescein diacetate (CMFDA) (CellTracker, Invitrogen, Molecular Probes, Oregon, USA) at a final concentration of 0.1 µg/ml and cultured at 7.5×10^5^ cells per well in 96-well plates (Falcon, Labware BD Biosciences, Oxnard, CA, USA) in complete RPMI 1640 media (GIBCO/BRL, Invitrogen, Gaithersburg, CA, USA) supplemented with 10% fetal calf serum (FBS, Hyclone, South Logan, UT, USA), L-glutamine (2 mM) and 1% penicillin/streptomycin. The cells were stimulated with human CpG oligodeoxynucleotides (Hycult Biotechnology, Uden, The Netherlands) at a concentration of 5 µg/ml and supplemented with interleukin 2 (IL-2) (25 IU/mL, Sigma Aldrich, St Louis, MO, USA). IL-2 was added to the cultures because we found that it improved cell survival in cryopreserved pathological samples without altering cell function. The cells were incubated for 4 and 7 days at 37°C in a humidified atmosphere containing 5% CO_2_. In parallel, basal PBMC cultures were set up by incubating PBMC from the same individual with complete medium plus IL-2 as a control. Cell proliferation was assessed after 4 and 7 days of CpG stimulation by flow cytometry using a FACSCanto II (Becton and Dickinson, Sunnyvale, CA, USA) and PC FACSDiva software (BD Biosciences, San Jose, CA, USA). Fifty thousand events per sample were analyzed.

### Flow cytometric analysis (FACS)

After 4- and 7-days of CpG stimulation, PBMC were harvested from the culture plates and washed by centrifugation at 1200 rpm for 5 minutes at room temperature (RT) in wash buffer (2% FBS in PBS). To identify B cell subsets, single-cell suspensions were stained on day 0 and after 4–7 days of stimulation with CpG or incubation with IL-2 alone with the appropriate combination of the following directly conjugated monoclonal antibodies (MoAb): CD19-Alexa Fluor 700 (1∶90 dilution; BD Biosciences, San Diego, CA, USA), CD38-PECy7 (1∶90 dilution; BD Biosciences), CD24-FITC (1∶10 dilution, BD Biosciences), CD27-PE (1∶20 dilution; BD Biosciences) and IgM-Alexa Fluor 647 (1∶400 dilution; Jackson ImmunoResearch, West Baltimore, Pike, PA, USA). The cells were incubated for 20 minutes in the dark at 4°C. After labeling, the cells were washed by centrifugation at 1200 rpm for 5 minutes at 4°C in wash buffer. Data were acquired on a FACSCanto II. Flow cytometry profiles were analyzed using FACSDiva software (BD Biosciences, San Jose, CA, USA). Dead cells were excluded from the analysis by side/forward scatter gating. A minimum of fifty thousand gated events on living cells were collected per data set.

### Statistical analysis

A Fisher’s exact test was computed for 2×2 tables as first step for analysis of all data sets related to healthy control C1858T *PTPN22* carrier and non-carrier populations and T1D C1858T *PTPN22* carrier and non-carrier populations. The normal distribution of values for healthy control C1858T *PTPN22* carrier and non-carrier populations and T1D C1858T *PTPN22* carrier and non-carrier populations was tested using the Kolmogorov-Smirnov test (KS-test). Group comparisons between the healthy control and the T1D populations were performed using one-way analysis of variance (one-way ANOVA) with Bonferroni’s multiple comparison test when the KS test was not significant, one-way ANOVA with Kruskal-Wallis test and Dunn’s multiple comparison test when the KS-test was significant. Two tailed Student *t* test was used to compare C/C and C/T *PTPN22* subjects; the unpaired *t* test with Welch’s correction was applied if the KS test was not significant, while Mann-Whitney test if the KS test was significant. The results were analyzed using GraphPad Prism software version number 5 (San Diego, CA, USA). A result with *p*<0.05 was considered statistically significant.

## Results

### Study population

The study population was composed of 26 T1D patients and 45 healthy controls. Of the 26 patients, 11 were carriers of the C1858T *PTPN22* polymorphism, and 15 were non-carriers. All patients were recruited during long-term disease. Of the 45 healthy controls, 10 were carriers of the variant, and 35 were non-carriers.

The mean actual age of the T1D patients who were non-carriers of the C1858T *PTPN22* polymorphism was 19.8 years (ranging from 11 to 30 years; 4 males, 11 females) (Table 1). The mean age at disease onset was 8.5 years (ranging from 3 to 19 years), and the mean duration of disease was 11.2 years (ranging from 4 to 19 years). The mean actual age of T1D patients who were carriers of the C1858T polymorphism was 18.09 years (ranging from 11 to 25 years; 6 males, 5 females) (Table 1). The mean age at disease onset was 7.3 years (ranging from 3 to 15 years), and the mean duration of disease was 10.7 years (ranging from 4 to 18 years). The mean age of the controls was 23 years (ranging from 18 to 30 years). The demographic information and clinical characteristics of patients are shown in Table 1.

**Table 1:** Clinical characteristics of T1D patients.

Patient	Gender	Age of disease onset	Actual age	Duration of disease	Associated diseases	*PTPN22* genotype	Basal percentage of transitional B cells
**AC**	F	4	15	11	AT	C/C	9.4
**SD**	M	7	11	4	AT	C/C	9.1
**VG**	F	4	15	11	CD	C/C	21.8
**AR**	M	19	30	11	AT; CD	C/C	12.2
**GR**	F	9	19	10	AT; vitiligo	C/C	3.4
**LI**	F	5	19	14	AT	C/C	9.4
**NA**	F	3	22	19	AT	C/C	6.8
**DV**	M	16	28	12	AT	C/C	5.3
**CL**	F	8	20	12	AT; AG	C/C	8.2
**GF**	F	8	16	8	obesity	C/C	1.5
**MF**	F	5	16	11	–	C/C	4.8
**SB**	F	3	14	11	AT; CD	C/C	4.3
**PG**	F	15	27	12	–	C/C	7.2
**AC**	M	13	26	13	CD	C/C	4.6
**GR**	F	9	19	10	AT	C/C	8.3
**BC**	F	15	25	10	AT	C/T	10.4
**SC**	M	7	11	4	CD	C/T	12.3
**FC**	M	3	15	12	CD	C/T	27.5
**SC**	M	4	15	11	CD	C/T	7.9
**DS**	M	14	25	11	AT	C/T	12.4
**EA**	F	8	16	8	–	C/T	10.9
**VB**	F	4	14	10	AT	C/T	11.1
**GT**	F	8	18	10	AT	C/T	14.1
**PH**	M	7	25	18	–	C/T	14.5
**PG**	M	4	18	14	AT	C/T	12.3
**FS**	F	7	17	10	–	C/T	10.8

AT: autoimmune thyroiditis; CD: celiac disease; AG: autoimmune gastritis.

Statistical analysis of basal transitional B cell percentages in C/C and C/T patients: KS-test p>0.10; unpaired T-test with Welch’s correction p = 0.03.

The T1D patients presented associated autoimmune manifestations (Table 1). Autoimmune gastritis was confirmed by the presence of PCA Abs. In addition to T1D, 15 patients developed autoimmune thyroid disease, i.e., Hashimoto’s thyroiditis (APS3v), confirmed by the presence of circulating Tg and TPO Abs and by an echographic pattern of diffuse hypoechogenicity, and 1 of these patients also presented vitiligo. In addition to T1D, 7 patients were affected by celiac disease, as confirmed by the presence of tTGA Abs at diagnosis.

### Altered B cell compartment in individuals heterozygous for the C1858T *PTPN22* variant in basal conditions (day 0)

The B cell phenotype of the PBMC was analyzed at day 0 for 32 healthy individuals and 23 patients affected by T1D. Of the 32 normal controls, 22 individuals had the common variant, and 10 were heterozygous for the C/T *PTPN22* variant. Within the population of T1D patients, 14 were wild type, and 9 were heterozygous for the C/T *PTPN22* variant.

**Figure 1:**
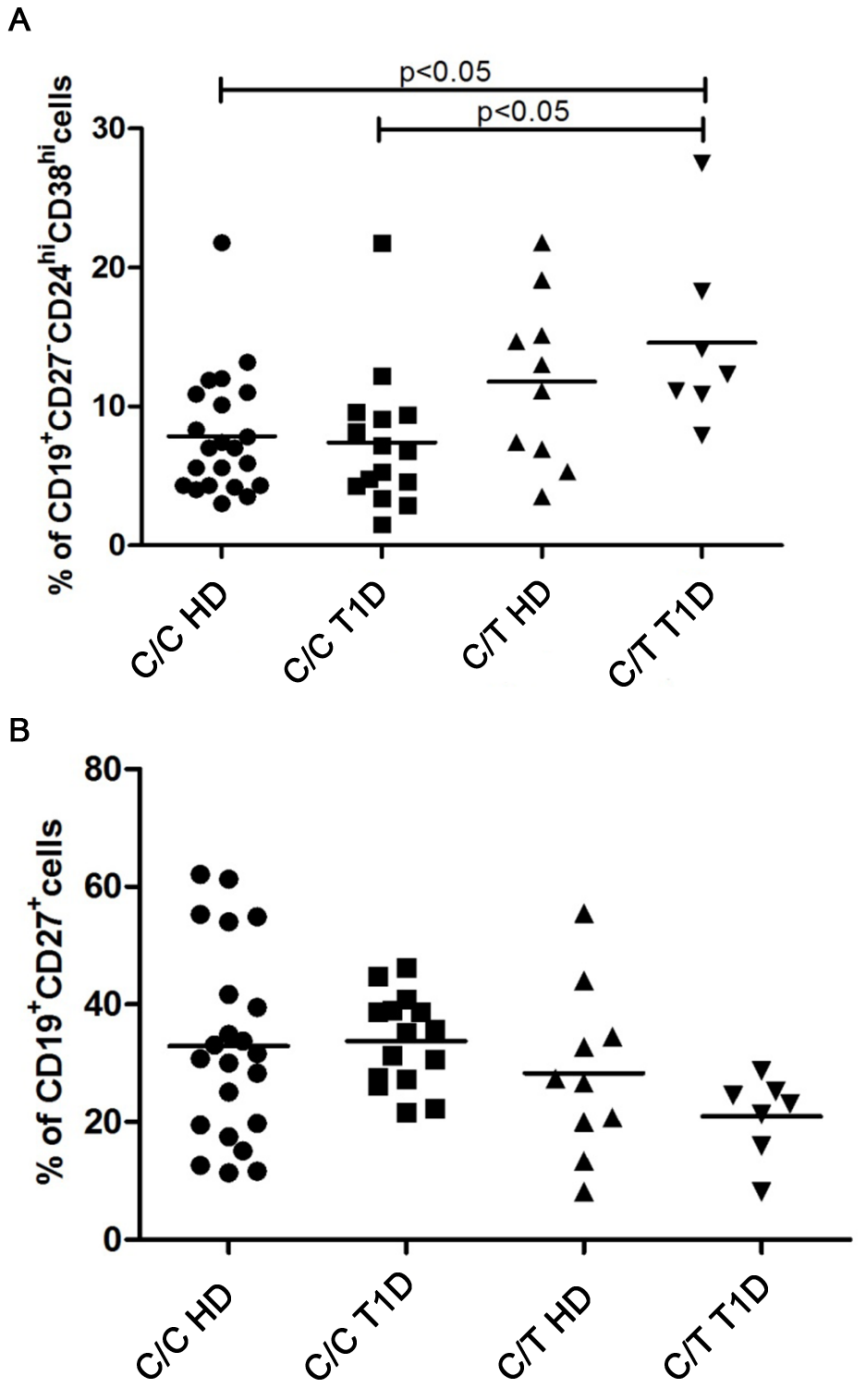
Altered B cell compartment in C1858T healthy controls and patients. Previously frozen PBMC from control C/T and C/C subjects and from C/T T1D and C/C patients were stained with antibodies to CD19, CD27, CD24 and CD38 and analyzed by FACS to determine the relative frequencies of transitional (**A**) and memory B cells (**B**) under basal conditions (day 0). In both, **A** and **B** graphs, horizontal lines represent the mean frequency and each symbol represents an individual.

Although the frequency and absolute number of peripheral CD19^+^ B cells did not differ between the two groups of controls (C/C and heterozygous for the C/T variant), the two groups of T1D patients (C/C and heterozygous for the C/T variant) or the four groups of healthy subjects and patients, the percentage of transitional B cells, defined as CD19^+^CD27^−^CD24^hi^CD38^hi^, was significantly increased in individuals who were heterozygous for the C/T variant (n = 36 C/C subjects, n = 19 C/T subjects) (KS-test p>0.10; unpaired *t* test with Welch’s correction p = 0.0046) [Figure S1A]. The percentage of transitional B cells was also significantly increased in the T1D patients who were heterozygous for the variant compared to the C/C healthy controls (*p*<0.05) and in the T1D patients heterozygous for the variant compared to the C/C T1D patients (KS-test p>0.10; one-way ANOVA with Bonferroni’s multiple comparison test p = 0.0075) (Figure 1A, Figure S2).

A statistically significant reduction in the percentage of memory B cells (CD19^+^CD27^+^) was observed in individuals heterozygous for the C/T *PTPN22* variant (both healthy individuals and T1D patients) compared to C/C individuals (KS-test p>0.10; unpaired t test with Welch’s correction p = 0.0359).(Figure S1B). There was no statistically significant decrease of memory B cells in C/C patients, patients harboring the variant and healthy heterozygous individuals compared to C/C healthy controls (KS-test p>0.10; one-way ANOVA p = 0.1410) (Figure 1B, Figure S2).

### Altered B cell phenotype composition of the PBMC of individuals carrying the C1858T *PTPN22* variant after 4 days of CpG stimulation

The B cell phenotypes of the PBMC were analyzed after 4 days of CpG stimulus for 17 healthy individuals and 23 T1D patients. Of the healthy subjects, 10 had the common *PTPN22* variant, and 7 were heterozygous for the C/T *PTPN22* heterozygous variant. Within the T1D population, 12 were C/C, and 5 were heterozygous for the C/T variant.

**Figure 2:**
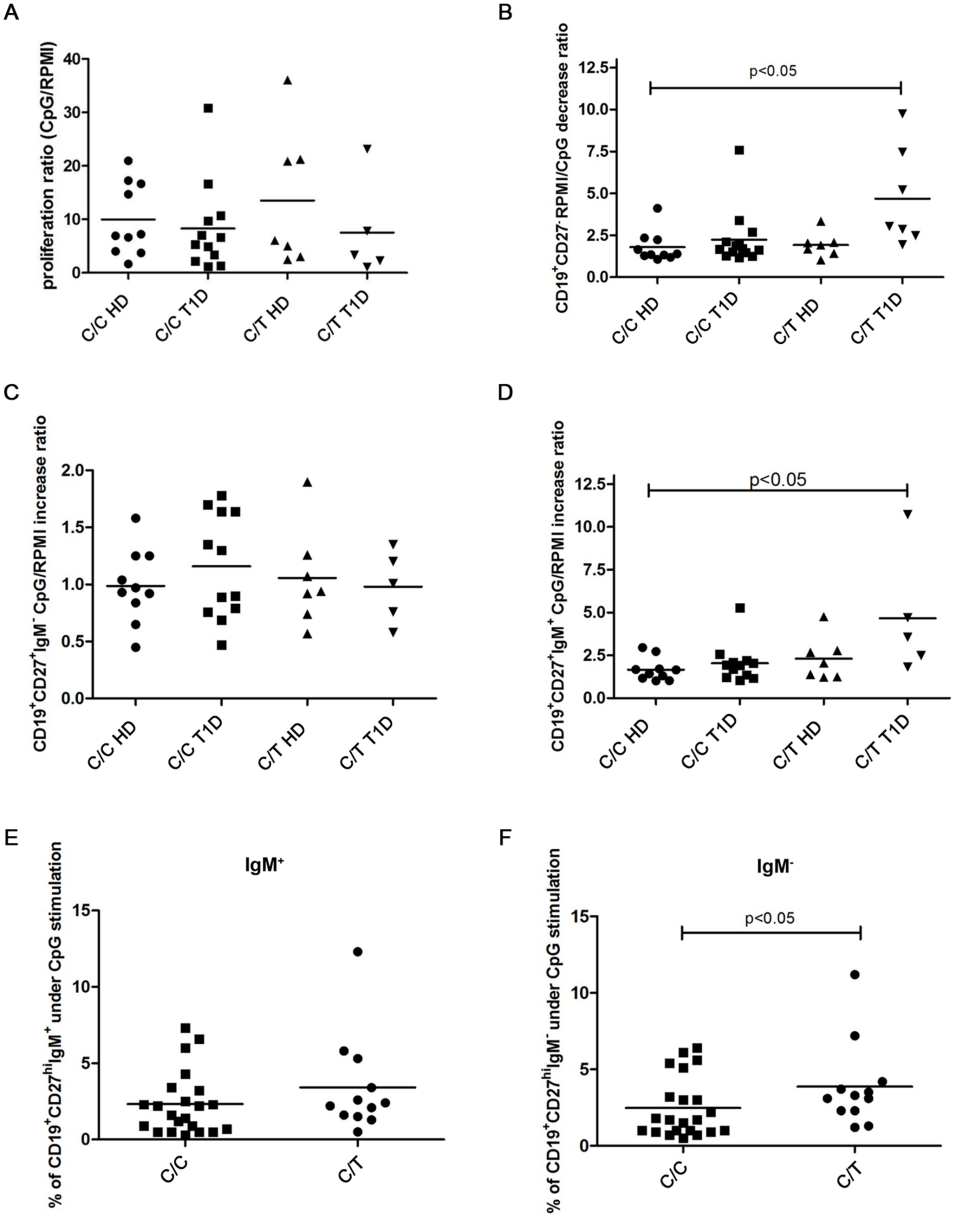
B cell phenotype after 4 days of CpG stimulation. Ratio of proliferation of CpG stimulated over unstimulated CMFDA-labeled CD19^+^ cells in the four different categories of subjects (**A**). Ratio of mature B cell percentages (**B**) in unstimulated compared to CpG-stimulated PBMC, ratio of switched memory B cell (**C**) and of IgM^+^ memory B cell percentages (**D**), calculated as CpG-stimulated over unstimulated PBMC, in T1D patients carrying or non carrying the C/T *PTPN22* variant and in non-carrier C/C and C/T healthy controls. Percentage of plasma cells from IgM^+^ (**E**) and IgM^−^ (**F**) memory B cells in the PBMC of individuals heterozygous for the C/T *PTPN22* variant compared to non-carriers.

After 4 days of stimulation with CpG, no significant difference in the proliferative response (calculated as ratio of proliferation of CD19^+^ cells over unstimulated cells) was observed between the PBMC obtained from individuals carrying the C/T *PTPN22* variant and the cells obtained from C/C individuals (KS-test p<0.05; Mann Whitney test p = 0.9569) (Figure S3). Furthermore, no differences in the proliferative responses were observed among the four different categories of subjects (KS-test p>0.10; one way ANOVA p = 0.6411) (Figure 2A).

Regarding the characterization of the B cell phenotype, a significant decrease in mature B cells (CD19^+^CD27^−^) was observed in heterozygous C/T individuals compared to C/C individuals (Figure S4). This decrease was expressed as the ratio of mature B cell percentages in the unstimulated over the CpG-stimulated PBMC (KS-test p<0.05; Mann-Whitney test p = 0.02) (Figure S4, Figure S5A). Within the four categories of individuals, the ratio of mature B cell percentages in unstimulated over CpG-stimulated PBMC was significantly increased in patients carrying the C/T *PTPN22* variant than in C/C healthy controls (KS-test p<0.05; one way ANOVA with Kruskal Wallis test and Dunn’s multiple comparison test p = 0.01) (Figure 2B, Figure S4, Figure S6).

There was no significant increase in the ratio of switched memory B cell (CD19^+^CD27^+^IgM^−^) percentages following CpG stimulation compared to the unstimulated cells among the four categories of individuals (KS-test p>0.10; one-way ANOVA p = 0.73) or between the heterozygous C/T versus C/C groups (KS-test p>0.10; unpaired t test with Welch’s correction p = 0.6863) (Figure 2C, Figure S4, Figure S5B).

When analyzing the IgM memory B cells, the ratio of IgM^+^ memory B cell (CD19^+^CD27^+^IgM^+^) percentages following CpG stimulation over unstimulated cells tended to significantly increase in T1D patients harboring the C/T *PTPN22* variant compared to C/C individuals (KS-test p<0.05; one way ANOVA with Kruskal-Wallis test and Dunn’s multiple comparison test p = 0.063) (Figure 2D, Figure S4, Figure S7). The ratio of IgM^+^ memory B cell percentages following CpG stimulation over unstimulated cells was also statistically significant between the groups of individuals heterozygous for the C/T variant compared to the C/C individuals (KS-test p<0.05; Mann-Whitney test p = 0.038). (Figure S5C).

When we examined the differentiation of IgM^+^ and class-switched memory B cells into antibody secreting cells, no statistically significant increase of the percentages of IgM^+^ plasma cells (CD19^+^CD27^hi^IgM^+^, PC) after four days of CpG stimulation in individuals that were heterozygous for the C/T *PTPN22* variant than in C/C individuals was detected (KS-test p<0.05; Mann-Whitney test p = 0.255) (Figure 2E). However, the overall increase in the percentage of IgM^−^ plasma cells (CD19^+^CD27^hi^IgM^−^) in individuals heterozygous for the C/T *PTPN22* variant was statistically significant compared to C/C individuals (KS-test p<0.05; Mann-Whitney test p = 0.038) (Figure 2F, p<0.05). Results were expressed only as percentages after CpG stimulation since the basal percentages of PC before CpG stimulation are always negligible.

### Altered B cell phenotype composition in PBMC of individuals carrying the C1858T *PTPN22* variant after 7 days of CpG stimulation

The phenotype of the B cells from the PBMC of 14 healthy individuals and 16 T1D patients was analyzed after 7 days of CpG stimulation. Of the healthy subjects, 9 were C/C, and 6 were heterozygous for the C/T *PTPN22* variant. Within the T1D population, 8 patients were C/C, and 8 were heterozygous for the risk variant.

**Figure 3:**
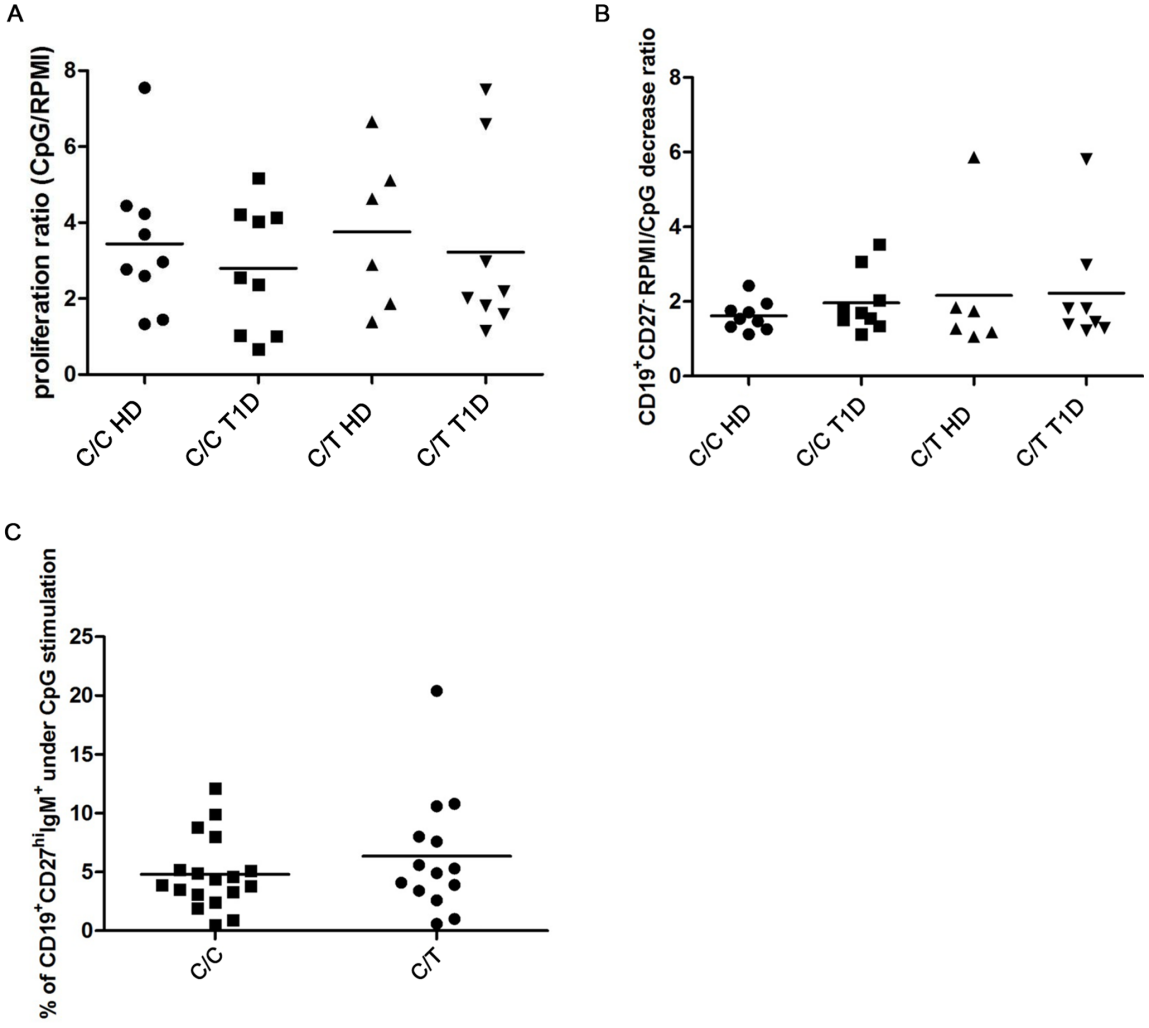
B cell phenotype after 7 days of CpG stimulation. Ratio of proliferation of CpG stimulated CMFDA-labeled CD19^+^ cells over unstimulated cells among the four different categories of subjects included in our study (**A**) Ratio of mature B cell percentages (**B**), calculated as unstimulated over CpG-stimulated PBMC, in T1D patients carrying or non carrying the C/T *PTPN22* variant and in non-carrier C/C and C/T healthy controls. Percentages of plasma cells from IgM^+^ memory B cells (**C**) in individuals carrying the C/T *PTPN22* variant compared to the C/C subjects.

After 7 days of stimulation with CpG, no difference in the proliferative response (calculated as ratio of CD19^+^ cells over unstimulated cells) was observed between PBMC obtained from individuals carrying the C/T *PTPN22* variant and in C/C individuals (KS-test p<0.05; Mann Whitney test p = 0.7757) (Figure S8). Furthermore, no difference in the proliferative response was observed among the four different categories of subjects, including C/C healthy controls, individuals heterozygous for the C/T *PTPN22* variant, C/C T1D patients and T1D patients heterozygous for the C/T *PTPN22* variant (KS-test p>0.10; one way ANOVA p = 0.6878) (Figure 3A).

The percentages of mature B cells observed in heterozygous C/T individuals compared to C/C individuals decreased without statistical significance (KS-test p<0.05; Mann-Whitney test p = 0.83) (Figure S9). The decrease was calculated as the ratio of mature B cells in unstimulated over CpG-stimulated PBMC. Within the four categories of individuals, the ratio of mature B cell percentages in unstimulated compared with CpG-stimulated PBMC increased without statistical significance in patients that possessed the C/T *PTPN22* variant than in C/C T1D patients or healthy controls (KS-test p<0.05; one way ANOVA with Kruskal-Wallis test p = 0.939) (Figure 3B, Figure S10).

There was no significant increase in the ratio of switched memory B cells following CpG stimulation compared to unstimulated cells across the four categories of individuals or between the heterozygous and C/C groups (data not shown). Similarly, there was no significant difference in the percentages of IgM^+^ memory B cells following CpG stimulation or unstimulated cells in individuals who were heterozygous for the risk variant compared to C/C individuals, and no differences in these percentages were detected across the four categories of subjects (data not shown).

We also found that there was no difference in the percentages of IgM^−^ plasma cells in heterozygous C/T carriers than in C/C individuals, and the ratio of percentages in CpG treated samples over unstimulated samples among the four categories of subjects was the same (data not shown). The percentages of IgM^+^ plasma cells did not change significantly and had a high standard deviation (KS-test p<0.05; Mann-Whitney test p = 0.36) (Figure 3C). We found that after 4 days of stimulation, the switched memory B cells tended to differentiate into plasma cells more precociously than IgM^+^ memory B cells.

## Discussion

In unraveling the potential pleiotropic effects of the Lyp variant in the autoimmunity process, similar to what has been reported by Habib and colleagues [20], we first confirmed that the frequency and absolute number of peripheral CD19^+^ B cells did not differ between C/C healthy subjects and those heterozygous for the C/T *PTPN22* variant (data not shown). Under basal conditions, we found that the presence of the variant resulted in a significantly increased percentage of transitional B cells in all heterozygous individuals, including both healthy controls and T1D patients, in heterozygous T1D patients compared to C/C healthy controls and in C/T compared to C/C T1D patients. Habib and colleagues [20] observed that the percentage of transitional B cells was higher in healthy subjects carrying the variant than in C/C controls. They found that the frequencies of both transitional and naïve anergic B cells were significantly increased in T1D patients than in C1858C controls. A comparison of paired T1D subjects by *PTPN22* genotype found that heterozygosity for the C1858T allele was not associated with the further expansion of transitional and anergic B cells. Similarly, the naïve and memory B cell profiles of T1D subjects paralleled C1858T healthy controls because there was an expansion in the naïve B cell subset and a reduction in the memory B cell pool in T1D patients, irrespective of the T cell pool. However, by examining a separate group of age-matched C1858C T1D and T1D patients homozygous for the C1858T *PTPN22* variant, a modest trend toward increased transitional and naïve B cells was found in subjects that were homozygous for the C1858T *PTPN22* variant, suggesting that the variant contributes to the diabetic phenotype [20]. These divergent findings may be due to the selection of the population sample in the two studies. In agreement with our data, both investigations suggest that in individuals carrying the variant, a large pool of autoreactive B cells enters and/or survives within the total B cell compartment and contributes to the breakdown of peripheral tolerance. In subjects carrying the Lyp R620W variant, this breakdown could promote the onset of autoimmune pathologies through the internalization and presentation of autoantigens to T lymphocytes (*vide infra*) [21], [38].

Our original approach was to further explore the effect of the Lyp variant on TLR9 CpG-induced stimulation of switched and IgM^+^ memory B cells and on plasma cell differentiation [35].

We found that CpG stimulation did not result in differences in the proliferative responses among the analyzed groups. The presence of the risk variant was associated with a reduction in the mature B cells in heterozygous C/T individuals compared to C/C healthy controls after 4 and 7 days of CpG stimulation and in heterozygous patients compared to C/C healthy controls after 4 days. There was no significant increase in the percentages of switched memory B cells induced after 4 and 7 days of CpG stimulation.

Remarkably, after 4 days of CpG stimulation, the IgM^+^ memory B cells were significantly increased in heterozygous T1D patients compared to C/C individuals and in the groups of individuals who were heterozygous for the variant compared to C/C individuals.

In non-organ-specific autoimmune disorders such as SLE, autoantibodies are a hallmark of disease and cause tissue and organ-damage [39]. Accumulating evidence thus suggests that stimulation of TLRs, i.e., TLR9, can incite autoimmunity in humans, and infections may play a role in triggering disease relapses [40]. In organ-specific T cell-mediated autoimmune disorders, such as T1D, CD4^+^ and CD8^+^ T lymphocytes are the major players in the destruction of pancreatic beta cells [41]. However, the role of B cells in T1D pathogenesis (*vide supra*) in humans remains unclear [41]. Experimental evidence suggests that the NOD strain is highly resistant to T1D when it is rendered deficient in B cells [27], [32], [33], [42]. NOD B cells, particularly transitional, B1, and marginal-zone B cells are the preferred antigen presenting cells (APC) for the priming and/or expansion of diabetogenic autoreactive CD4^+^ T cells. Moreover, independent of their pathogenic role, B cells produce islet-related IgG autoantibodies as the earliest manifestation of the diabetogenic process [41]. Antibodies binding to beta cells before the development of insulitis primarily of the IgM isotype, are spontaneously secreted by B1a B cells in the absence of exogenous stimuli or T cell help [43]. NOD B1a B cells express high levels of TLRs and have a low threshold for innate immune activation. In humans, it has been suggested that IgM^+^ memory B cells may belong to a separate lineage of B cells generated through a T cell-independent mechanism [44] and may be functionally homologous to the mouse B-1a B cells.

The results of our investigation further demonstrate the role of innate immune mechanisms in T1D pathogenesis and identify the influence of the Lyp variant on the ability to produce autoreactive IgM memory B cells (also known as circulating marginal zone B cells) that lead to antibody production following infections.

As observed in previous studies regarding the B cell phenotype (*vide supra*) [21], [22], the altered B cell homeostasis and TLR9-driven response that was reported in C/T T1D patients and in healthy controls emphasizes the potential effect of the variant prior to the clinical onset of autoimmunity in predisposed individuals.

Independent of the pathogenic influences that the Lyp variant can have in the progression of disease, our findings have potential translational implications. The increased TLR response in T1D patients harboring the *PTPN22* variant may be used to identify patients in whom the increased susceptibility to infections may place beta cell reservoirs at an increased risk when attacked by the expanded potential T cell-independent IgM memory B cells [44], [45]. If our results can be confirmed in extended population studies, the effects of the variant on the TLR response in T1D patients may become a predictive marker of a more severe outcome of disease and could forecast the necessity for future adjustments of the regimen of insulin therapy.

An alternative speculative hypothesis, to be unraveled in clinical setting, suggests that this subgroup of patients serves as potential candidates for B cell-depleting strategies as an alternative to T cell immunotherapies [46]. Remarkably IgG levels were in general unaffected, while IgM levels were reduced and remained lower in patients treated with anti-CD20 in a follow-up of 30 months [47], [48], [49]; however, the mechanisms and clinical implications of this phenomenon remain unclear.

Our results provide an additional demonstration of the effect of the Lyp variant on the development of innate and adaptive immune responses and will serve as the basis for future investigations into the relative contribution of *PTPN22* function in the pathogenesis and progression of T1D.

## Conclusions

T1D is an autoimmune disease caused by the destruction of pancreatic beta cells by autoreactive T cells. Among the genetic variants strongly associated with the disease, the Lyp variant alters not only the function of T cells but also that of B cells in both innate and adaptive immunity. In our study, the presence of the Lyp variant resulted in a significantly increased percentage of transitional B cells in heterozygous subjects (both T1D patients and controls) compared to C/C subjects. A significant reduction in the memory B cell population was also observed in the presence of the variant. After CpG stimulation, a significant increase in the IgM^+^ memory B cells was detected in heterozygous diabetics compared to C/C subjects and in the groups of individuals who were heterozygous for the variant compared to C/C individuals. In addition to the observation that the Lyp variant diminishes IFN production and IFN responses upon TLR stimulation, thus predisposing individuals with the variant to uncontrolled infections, the increased TLR9 response leading to expanded T cell-independent IgM^+^ memory B cells that was observed in our study will propel future investigations into the relative contribution of *PTPN22* function in the pathogenesis and progression of T1D.

## Supporting Information

Figure S1 **Altered B cell compartment in C1858T healthy controls and patients.** Percentage of transitional B cells (**A**) and of memory B cells (**B**) in individuals carrying the heterozygous C/T *PTPN22* variant (both healthy individuals and T1D patients) compared to C/C individuals.
(TIF)

Figure S2 **FACS gating strategy to analyze the baseline B cell phenotype.** Representative contour plot analysis of PBMC from a T1D patient heterozygous for C/T *PTPN22* (**A**) identifying transitional B cells as CD24^hi^CD38^hi^ gated on CD19^+^ cells and from a C/C healthy control (**B**). Values indicate the percentage of transitional (TRANS), mature (MT) and memory (MEM) CD19^+^ B cells. The patient shows an increased frequency of TRANS B cells.
(TIF)

Figure S3 **B cell proliferation after 4 days of CpG stimulation.** Proliferative response of CMFDA-labeled CD19^+^ cells (calculated as the ratio of CpG-stimulated over unstimulated cells) in the C/C and C/T *PTPN22* subjects.
(TIF)

Figure S4 **FACS gating strategy to analyze the B cell phenotype after CpG stimulation.** B cell phenotype analysis in the PBMC of a C/T *PTPN22* T1D patient carrier (**A**, **B**) and a C/C healthy control (**C**, **D**). Representative dot plot analysis showing the gate obtained for CD19^+^ cells in unstimulated PBMC (**A**, **C** upper panels) and of the gated B cell population showing the percentages of PC (CD19^+^CD27^hi^IgM^+^ and CD19^+^CD27^hi^IgM^−^), switched memory (SW M, CD19^+^CD27^+^IgM^−^), IgM^+^ memory B cells (IgM M, CD19^+^CD27^+^IgM^+^) and mature (MT, CD19^+^CD27^−^) cells (**A**, **C** bottom panels). Representative dot plots of the same cytometric analysis in CpG-stimulated PBMC (**B**, **D** upper and bottom panels). CpG induces a similar proliferative response of the overall B cell population in PBMC of both the patient and the control. CpG induction resulted in a lower increase in the percentages of SW M and IgM M cells and a lower reduction of the percentage of MT cells compared to unstimulated cells in the C/C control than in the C/T patient.
(TIF)

Figure S5 **B cell phenotype after 4 days of CpG stimulation.** Ratio of mature B cell percentages calculated as unstimulated over CpG-stimulated PBMC (**A**), of switched memory B (**B**) and of IgM^+^ memory B cell percentages (**C**) calculated as CpG-stimulated over unstimulated PBMC.
(TIF)

Figure S6 **Analysis of mature B cells**. **after 4 days of CpG stimulation.** The graph shows the same analysis of Figure 2B. Bars show median of values.
(TIF)

Figure S7 **Analysis of IgM^+^ memory B cells** **after 4 days of CpG stimulation.** The graph shows the same analysis of Figure 2D. Bars show median of values.
(TIF)

Figure S8 **B cell proliferation after 7 days of CpG stimulation.** Ratio of proliferation of CpG-stimulated over unstimulated CMFDA-labeled CD19^+^ cells in the C/C and C/T *PTPN22* subjects.
(TIF)

Figure S9 **Analysis of mature B cells after 7 days of CpG stimulation.** Ratio of mature B cell percentages in unstimulated over CpG-stimulated PBMC in individuals heterozygous for the C/T *PTPN22* variant compared to C/C individuals.
(TIF)

Figure S10 **B cell phenotype after 7 days of CpG stimulation.** Analysis of mature B cells. The graph shows the same analysis of Figure 3B. Bars show median of values.
(TIF)
