# Supplementary figures and images for: Altered B Cell Homeostasis and Toll-Like Receptor 9-Driven Response in Type 1 Diabetes Carriers of the C1858T PTPN22 Allelic Variant: Implications in the Disease Pathogenesis
Source: PLoS One. 2014 Oct 21;9(10):e110755. doi: 10.1371/journal.pone.0110755 (PMC4205012; doi:10.1371/journal.pone.0110755)

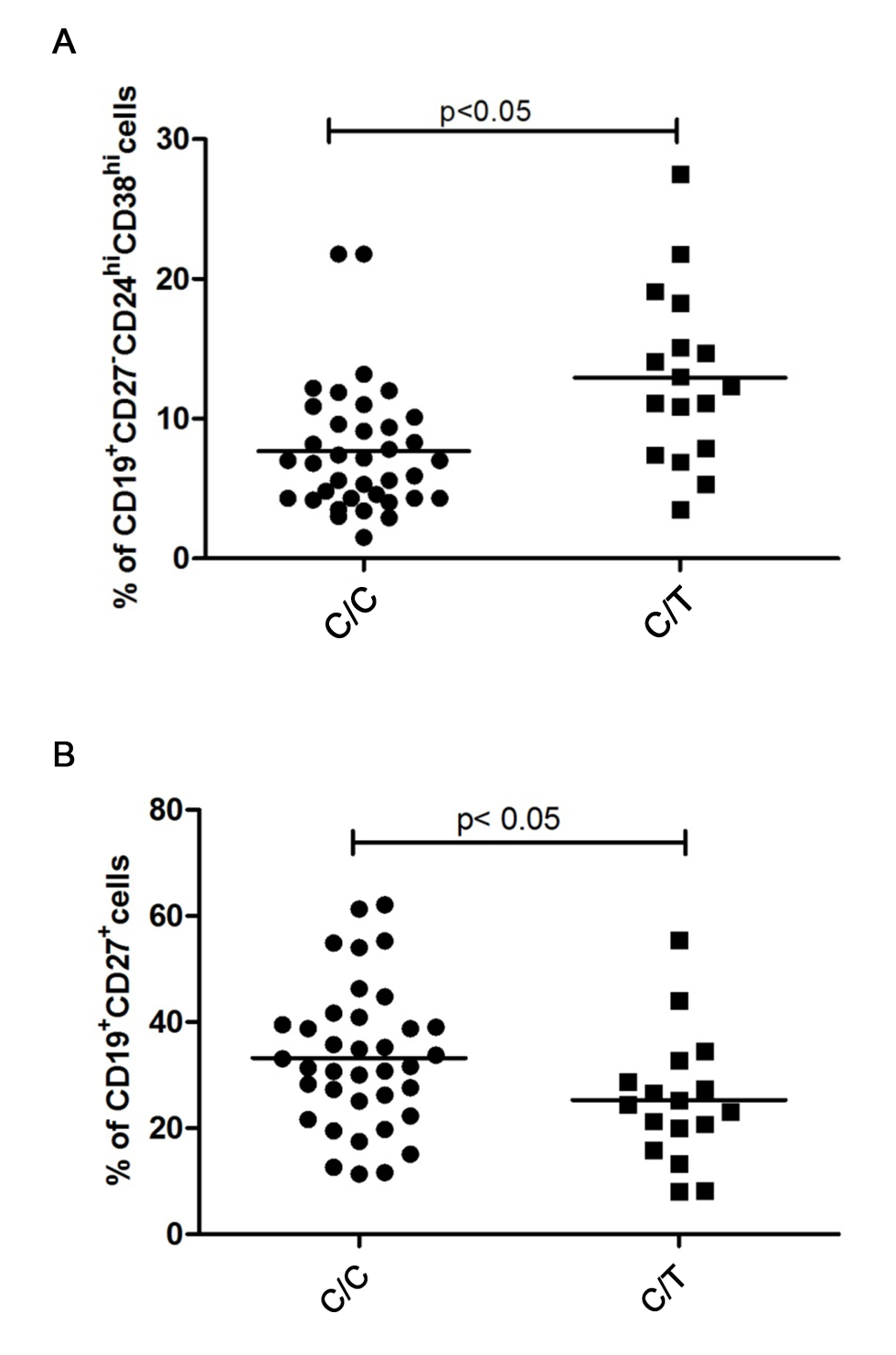

Supplement: Figure S1 — Altered B cell compartment in C1858T healthy controls and patients. Percentage of transitional B cells (A) and of memory B cells (B) in individuals carrying the heterozygous C/T PTPN22 variant (both healthy individuals and T1D patients) compared to C/C individuals. (TIF) [file pone.0110755.s001.tif]

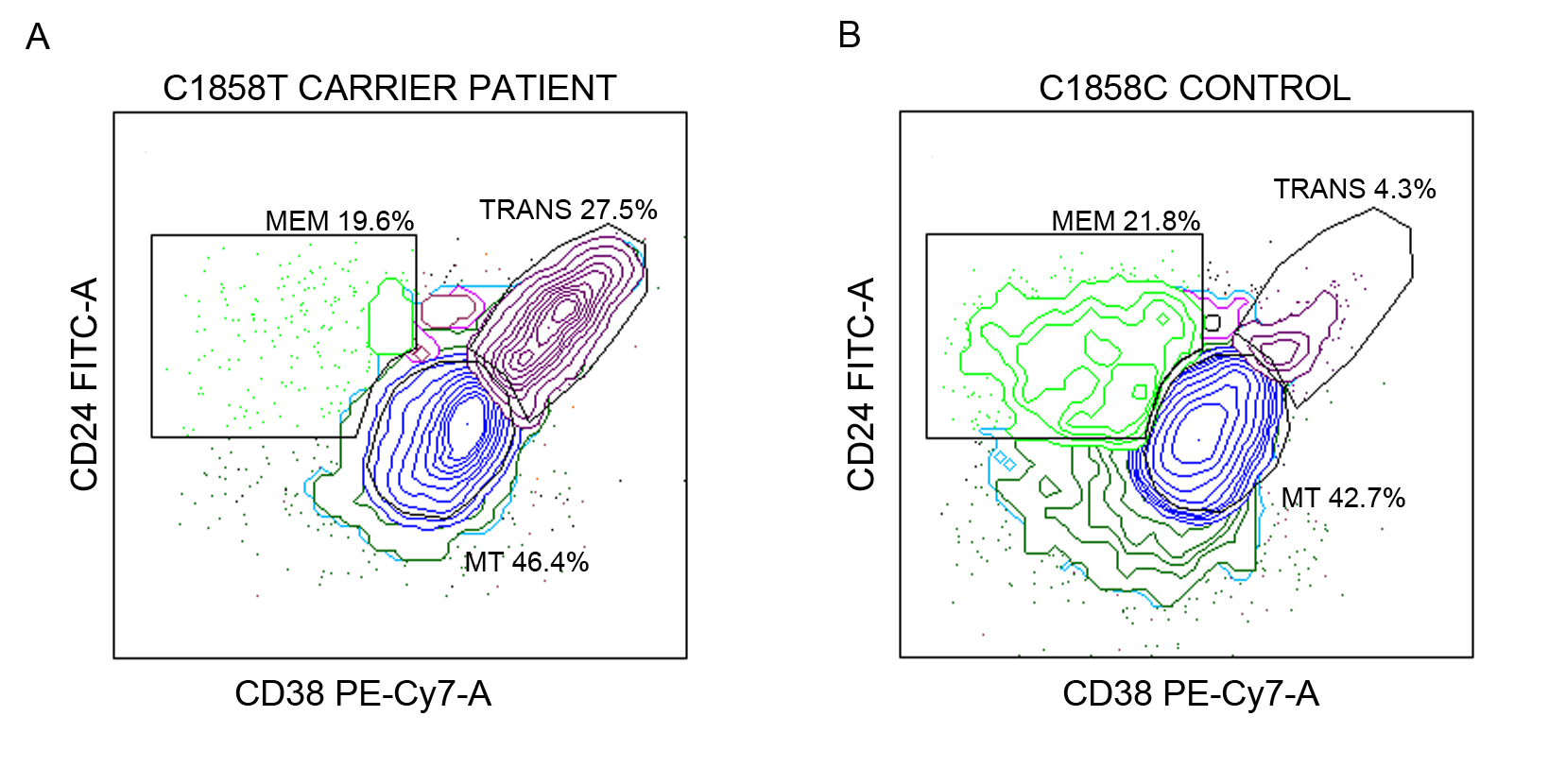

Supplement: Figure S2 — FACS gating strategy to analyze the baseline B cell phenotype. Representative contour plot analysis of PBMC from a T1D patient heterozygous for C/T PTPN22 (A) identifying transitional B cells as CD24hiCD38hi gated on CD19+ cells and from a C/C healthy control (B). Values indicate the percentage of transitional (TRANS), mature (MT) and memory (MEM) CD19+ B cells. The patient shows an increased frequency of TRANS B cells. (TIF) [file pone.0110755.s002.tif]

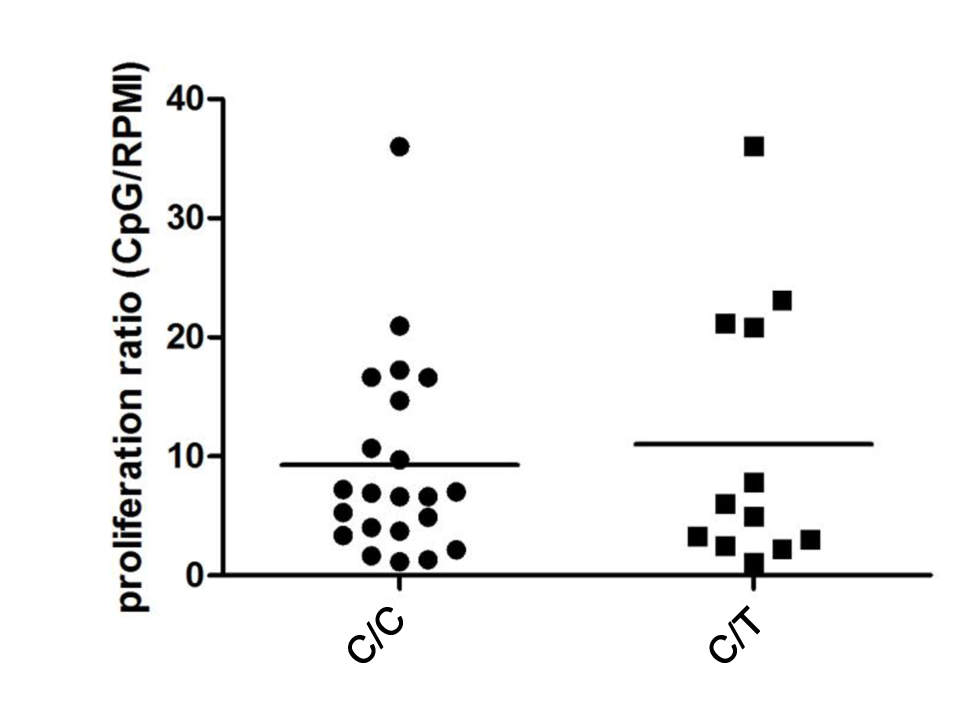

Supplement: Figure S3 — B cell proliferation after 4 days of CpG stimulation. Proliferative response of CMFDA-labeled CD19+ cells (calculated as the ratio of CpG-stimulated over unstimulated cells) in the C/C and C/T PTPN22 subjects. (TIF) [file pone.0110755.s003.tif]

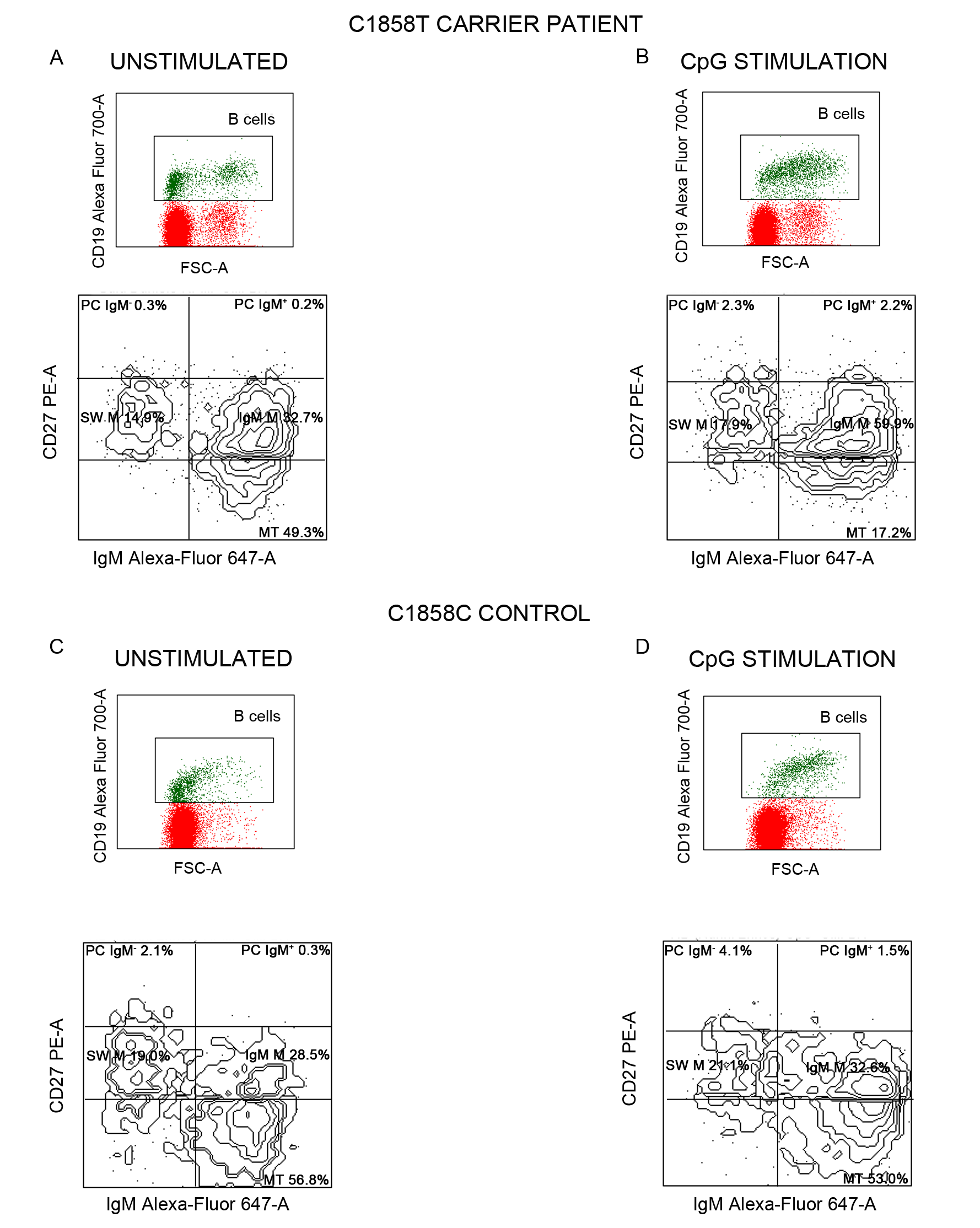

Supplement: Figure S4 — FACS gating strategy to analyze the B cell phenotype after CpG stimulation. B cell phenotype analysis in the PBMC of a C/T PTPN22 T1D patient carrier (A, B) and a C/C healthy control (C, D). Representative dot plot analysis showing the gate obtained for CD19+ cells in unstimulated PBMC (A, C upper panels) and of the gated B cell population showing the percentages of PC (CD19+CD27hiIgM+ and CD19+CD27hiIgM−), switched memory (SW M, CD19+CD27+IgM−), IgM+ memory B cells (IgM M, CD19+CD27+IgM+) and mature (MT, CD19+CD27−) cells (A, C bottom panels). Representative dot plots of the same cytometric analysis in CpG-stimulated PBMC (B, D upper and bottom panels). CpG induces a similar proliferative response of the overall B cell population in PBMC of both the patient and the control. CpG induction resulted in a lower increase in the percentages of SW M and IgM M cells and a lower reduction of the percentage of MT cells compared to unstimulated cells in the C/C control than in the C/T patient. (TIF) [file pone.0110755.s004.tif]

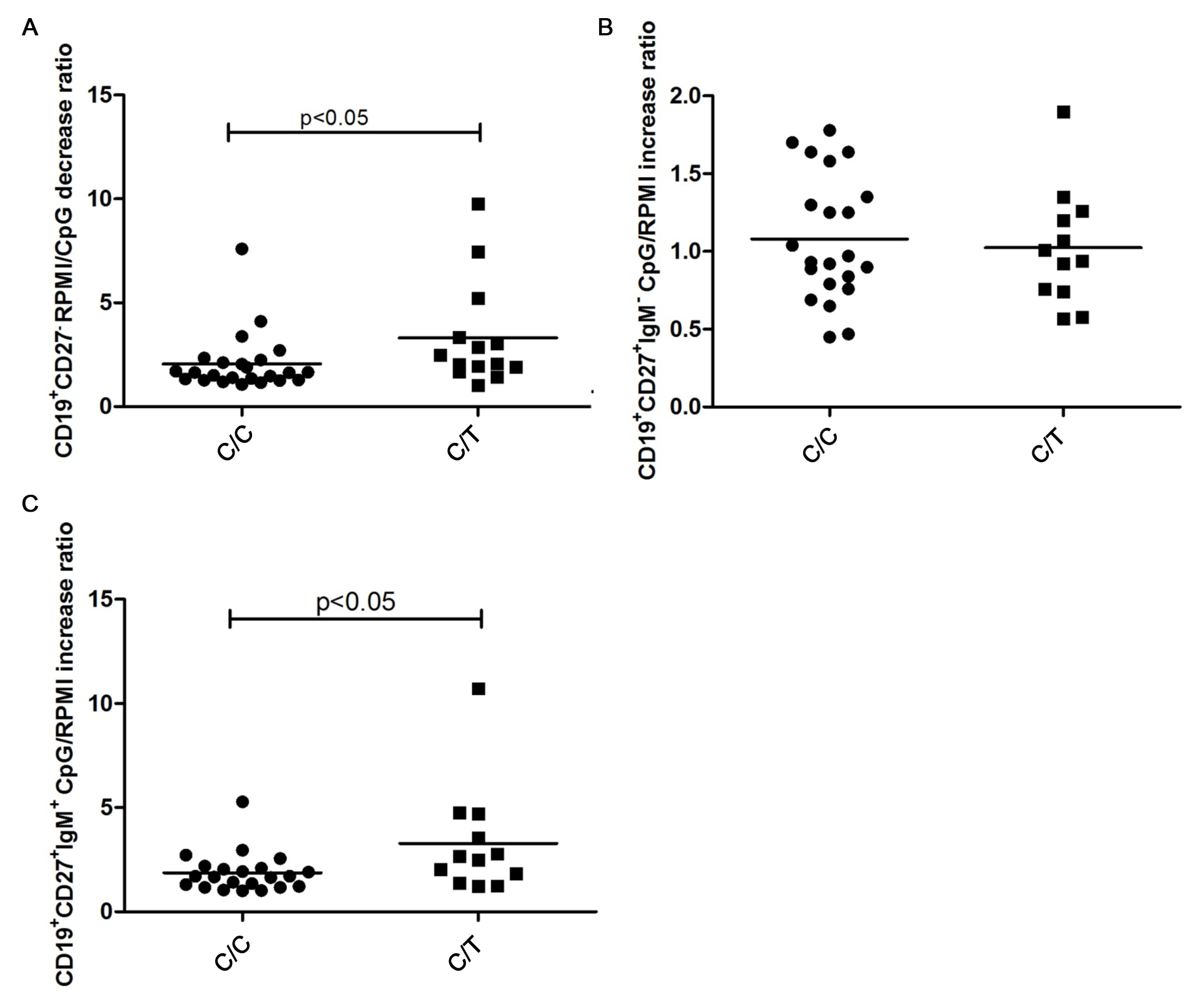

Supplement: Figure S5 — B cell phenotype after 4 days of CpG stimulation. Ratio of mature B cell percentages calculated as unstimulated over CpG-stimulated PBMC (A), of switched memory B (B) and of IgM+ memory B cell percentages (C) calculated as CpG-stimulated over unstimulated PBMC. (TIF) [file pone.0110755.s005.tif]

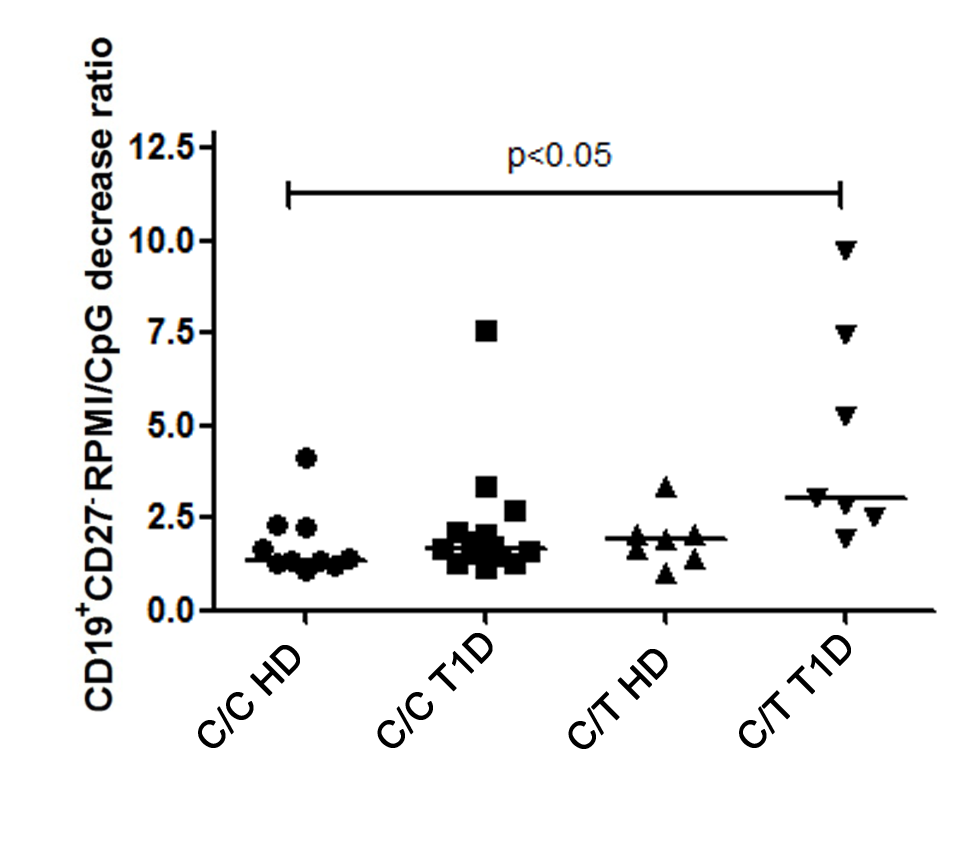

Supplement: Figure S6 — Analysis of mature B cells. after 4 days of CpG stimulation. The graph shows the same analysis of Figure 2B. Bars show median of values. (TIF) [file pone.0110755.s006.tif]

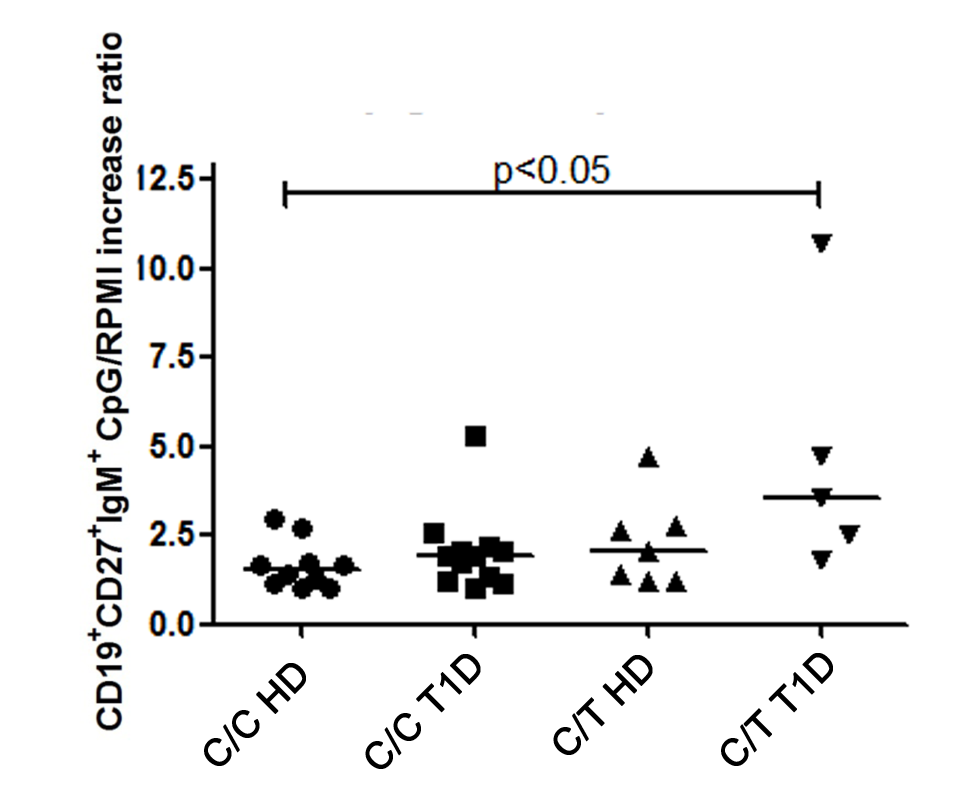

Supplement: Figure S7 — Analysis of IgM+ memory B cells after 4 days of CpG stimulation. The graph shows the same analysis of Figure 2D. Bars show median of values. (TIF) [file pone.0110755.s007.tif]

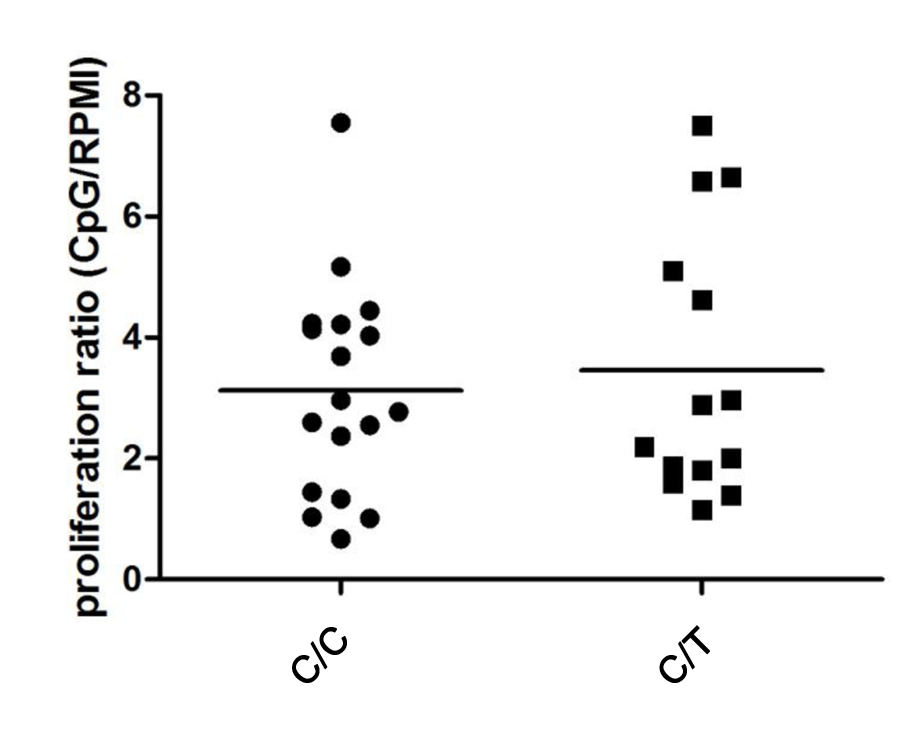

Supplement: Figure S8 — B cell proliferation after 7 days of CpG stimulation. Ratio of proliferation of CpG-stimulated over unstimulated CMFDA-labeled CD19+ cells in the C/C and C/T PTPN22 subjects. (TIF) [file pone.0110755.s008.tif]

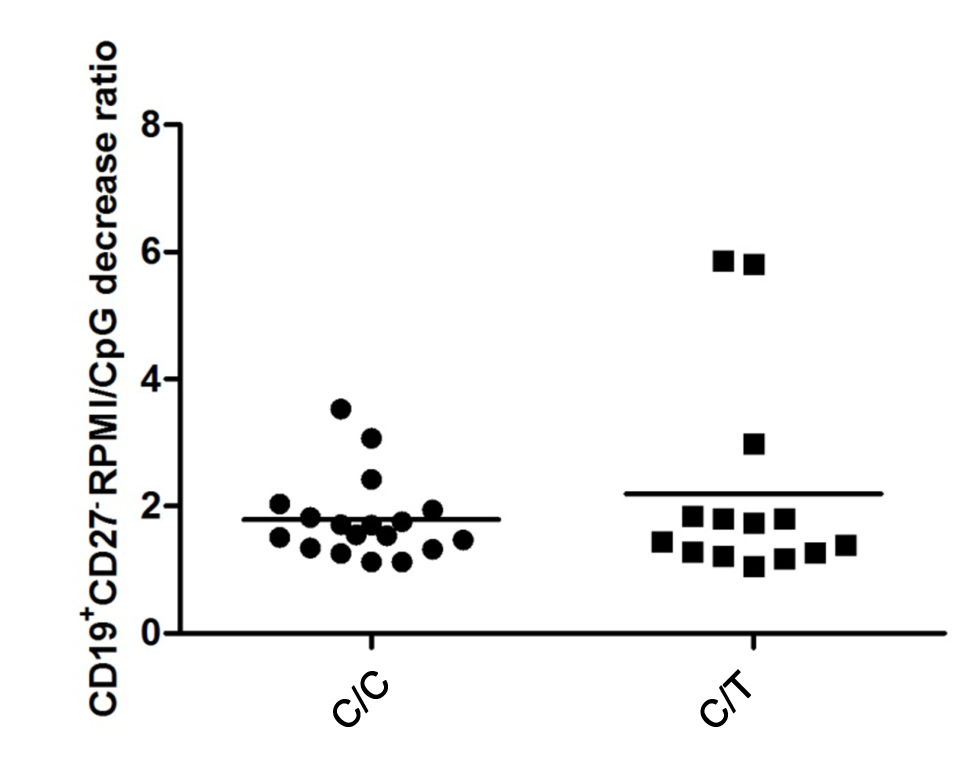

Supplement: Figure S9 — Analysis of mature B cells after 7 days of CpG stimulation. Ratio of mature B cell percentages in unstimulated over CpG-stimulated PBMC in individuals heterozygous for the C/T PTPN22 variant compared to C/C individuals. (TIF) [file pone.0110755.s009.tif]

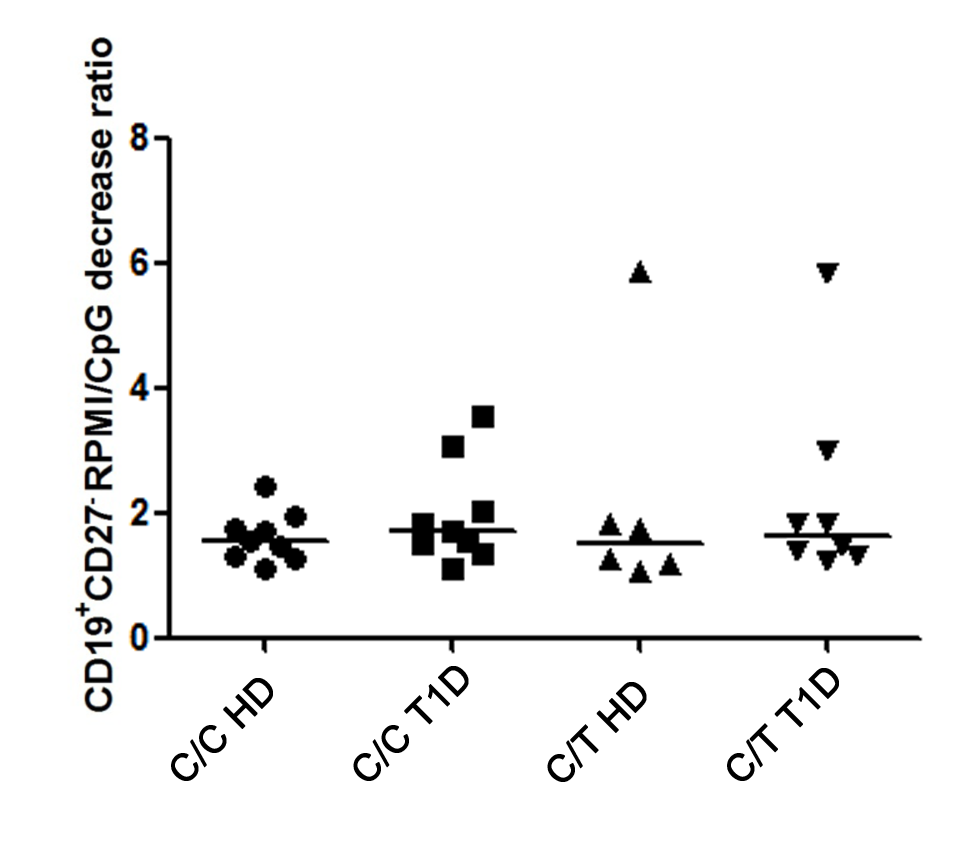

Supplement: Figure S10 — B cell phenotype after 7 days of CpG stimulation. Analysis of mature B cells. The graph shows the same analysis of Figure 3B. Bars show median of values. (TIF) [file pone.0110755.s010.tif]
